# Leukocyte telomere attrition in cognitive decline: associations with APOE genotype and cardiovascular risk factors

**DOI:** 10.3389/fnagi.2025.1557016

**Published:** 2025-04-15

**Authors:** Alexandre Guimarães de Almeida Barros, Thayana Oliveira Soares, Ariane Flávia Almeida Lage, Marco Túlio Gualberto Cintra, Jonas Jardim de Paula, Olívio Brito Malheiro, Antonio Eiras Falcão, Christiano Altamiro Coli Nogueira, Leandro Braz de Carvalho, Marco Aurélio Romano Silva, Debora Marques de Miranda, Bernardo de Mattos Viana, Daniela Valadão Freitas Rosa, Maria Aparecida Camargos Bicalho

**Affiliations:** ^1^Medical School of Universidade Federal de Minas Gerais, Belo Horizonte, Brazil; ^2^Hospital da Unimed Contorno Unimed-BH, Belo Horizonte, Brazil; ^3^Medical School of Universidade Estadual de Campinas, Campinas, Brazil

**Keywords:** telomere attrition, aging, Alzheimer’s disease, cognitive decline, neurodegenerative disease

## Abstract

Telomere shortening represents a fundamental mechanism of cellular aging potentially implicated in neurodegenerative processes. This study investigated the complex associations among leukocyte telomere length, cardiovascular risk profiles, and APOE polymorphisms in age-related cognitive decline. Through a cross-sectional analysis of 90 participants stratified by cognitive status into three groups: cognitively unimpaired (CU), mild cognitive impairment (MCI), and Alzheimer’s Disease (AD), we quantified relative telomere length using quantitative PCR, performed APOE genotyping and assessed cardiovascular risk factors. Quantitative analysis revealed significantly reduced telomere length in the AD group compared to CU and MCI groups. Multivariate regression analysis identified cognitive status as an independent predictor of telomere length (*β* = −0.468, *p* < 0.001). APOE ε4 carrier status showed higher prevalence in AD subjects as expected. Cardiovascular risk factors demonstrated no significant correlation with telomere length across cognitive groups. Our findings establish a robust association between telomere shortening and advanced cognitive impairment in AD, suggesting potential utility as a neurodegenerative biomarker. This relationship appears independent of traditional cardiovascular risk factors, highlighting the complexity of cellular aging mechanisms in neurodegeneration.

## Graphical abstract

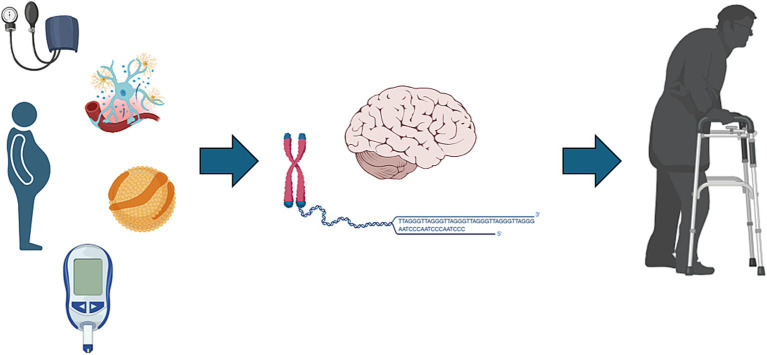

Summarizing the relationship between leukocyte telomere shortening and Alzheimer’s disease (AD). Telomere attrition, driven by mechanisms such as oxidative stress, chronic inflammation, and cellular senescence, contributes to neurodegeneration characteristic of AD pathology, including amyloid-β deposition and tau aggregation. Shorter telomeres are significantly associated with advanced cognitive impairment observed in AD patients. This highlights the potential of telomere length as part of a biomarker panel for cognitive decline and suggests telomere maintenance as a promising therapeutic target to mitigate neurodegeneration.

## Introduction

Aging is an irreversible biological process marked by progressive declines in physiological functions and an increased susceptibility to diseases, including neurodegenerative disorders such as Alzheimer’s disease (AD) (Kennedy et al., 2014; Melzer et al., 2020; López-Otín et al., 2013; López-Otín et al., 2023; Vaiasicca et al., 2024; Jothi and Kulka, 2024). As the primary risk factor for AD, aging is characterized by progressive cognitive decline, initially manifesting as mild cognitive impairment (MCI), which often progresses to dementia, primarily affecting memory (Knopman et al., 2021; Zvěřová, 2019; Kamatham et al., 2024; Testo et al., 2024). Aging and AD share common pathological features, including amyloid-*β* plaques, tau protein tangles, synaptic loss, and neuroinflammation, processes that commence several years before clinical symptoms appear. The overlap in these pathological mechanisms suggests shared biological pathways between aging and AD (Vaiasicca et al., 2024; Zvěřová, 2019; Savva et al., 2009); however, the precise mechanisms linking aging to AD remain incompletely understood.

Telomeres, repetitive DNA sequences protecting chromosome ends, serve as biological markers of cellular senescence and influences aging and age-related diseases (Blasco, 2005; López-Otín et al., 2023; Blackburn et al., 2015; Rossiello et al., 2022; Fani et al., 2019; Vaiserman and Krasnienkov, 2021). For instance, telomere attrition activates DNA Damage Response (DDR) pathways, culminating in cellular senescence, a hallmark of organismal aging (Dahse et al., 2020; López-Otín et al., 2023; Rossiello et al., 2022; Revy et al., 2023). Moreover, telomeres are susceptible to oxidative stress, accelerating their shortening and amplifying DDR. This response contributes to systemic inflammation through the senescence-associated secretory phenotype (SASP) (López-Otín et al., 2023; Rossiello et al., 2022; Revy et al., 2023). Recent studies implicate telomere attrition in the pathogenesis of AD through the promotion of cellular senescence and chronic inflammation associated with SASP (Fani et al., 2019; Yu et al., 2021; Rodríguez-Fernández et al., 2022; Levstek et al., 2020). In addition, the accumulation of sites of irreparable DNA damage response, named telomere-associated foci (TAFs), in hippocampal neurons, microglia, and oligodendrocyte progenitor cells is linked to neuroinflammation and cognitive decline, underscoring the potential role of telomere dysfunction in AD pathology (López-Otín et al., 2023; Rossiello et al., 2022; Fani et al., 2019; Levstek et al., 2020; Harley et al., 2024; Crocco et al., 2023; Guo and Yu, 2019). Although our study does not intend to explore the molecular mechanisms underlying the link between telomere aging and Alzheimer’s disease, the cited examples underscore the putative association between telomeres, aging, and AD, suggesting the potential of telomeres as biomarkers for AD (Daios et al., 2022).

Cardiovascular risk factors, including diabetes mellitus (DM), hypertension, and dyslipidemia, are established risk factors for cognitive decline and AD (Livingston et al., 2020). These conditions likely accelerate neurodegeneration by enhancing oxidative stress and chronic inflammation. Additionally, cardiovascular factors such as oxidative stress and inflammation are strongly associated with accelerated telomere shortening, which may contribute to cellular aging (Kennedy et al., 2014; Knopman et al., 2021; Livingston et al., 2020; Hua et al., 2020). The relationship between cardiovascular conditions and cognitive impairment suggests a potential mechanistic link that may also be reflected in telomere shortening, underscoring the importance of investigating cardiovascular health in the context of cognitive aging.

Physical exercise also plays a critical dual role, influencing both cognitive function and telomere biology (Sánchez-González et al., 2024; Eitan et al., 2014). Regular physical activity has been associated with longer telomere length, potentially mediated through reduced oxidative stress and inflammation, improved cardiovascular health, and enhanced neuroplasticity, which collectively contribute to cognitive preservation (Sánchez-González et al., 2024; Eitan et al., 2014; Sánchez-González et al., 2021; Sánchez-González et al., 2025). Therefore, lifestyle factors such as physical exercise warrant consideration when examining telomere length and cognitive function relationships. Although this study does not directly explore physical exercise, the analyzed body composition serves as a surrogate marker of the patients’ lifestyle and may reflect it, albeit with certain limitations.

Moreover, genetic factors such as the APOE ε4 allele significantly influence cardiovascular health and AD risk, likely via mechanisms involving oxidative stress, lipid metabolism disturbances, and inflammation (Eitan et al., 2014; Wikgren et al., 2012; Takata et al., 2012). Clarifying interactions between APOE genotype and telomere length could provide insights into individual susceptibility to AD pathology.

This study aims to investigate the relationships between relative leukocyte telomere length, cardiovascular risk factors, body composition, and APOE polymorphisms in cognitive decline contexts among older adults. Given the existing evidence linking cardiovascular risk factors, lifestyle, APOE genotype, and telomere attrition to cognitive impairment, exploring these interactions can enhance our understanding of the cellular aging mechanisms involved in AD development and validate telomere attrition as a potential biomarker for AD. The insights from this research may contribute to improved risk stratification and inform targeted interventions aimed at mitigating cognitive decline.

## Methods

### Study design and setting

This observational, cross-sectional study followed the Strengthening the Reporting of Observational Studies in Epidemiology (STROBE) guidelines (Supplementary Checklist; Von Elm et al., 2007). The study was conducted at the outpatient geriatric clinic of the University Hospital, Universidade Federal de Minas Gerais (UFMG), Belo Horizonte, Brazil. Participants were referred from primary care services and recruited through convenience sampling between March and December 2017. While a longitudinal approach would ideally evaluate changes in telomere length associated with cognitive decline, resource limitations and logistical constraints guided the selection of a cross-sectional design. The Ethics Committee of UFMG approved the study (CAAE 64362717.7.0000.5149), and all participants provided written informed consent.

### Participants, variables, and protocol

Inclusion criteria were age ≥ 60 years; clinical diagnosis of cognitively unimpaired (CU), mild cognitive impairment (MCI) or Alzheimer’s disease dementia (AD) status; neuropsychological and geriatric assessments performed within 6 months; complete anthropometric measurements, body composition analysis (DXA), and informed consent.

Exclusion criteria included psychiatric disorders (major depression, bipolar disorder, schizophrenia), other neurodegenerative diseases (Parkinson’s disease, dementia with Lewy bodies), vascular cognitive impairment, neoplasms, severe sensory deficits, or use of acetylcholinesterase inhibitors or memantine.

Briefly, participants underwent comprehensive geriatric and neuropsychological evaluations to establish cognitive and functional status, classified into CU, MCI, and AD groups following DSM-5 and McKhann et al. criteria. Assessments included Mini-Mental State Examination (MMSE), Clock Drawing Test, Verbal Fluency Test, CERAD Word List, Brief Cognitive Battery, Pfeffer Functional Activities Questionnaire, Clinical Dementia Rating (CDR), Geriatric Depression Scale, Neuropsychiatric Inventory, and DSM-5 criteria for major depressive disorder. Neuropsychological testing included the Mattis Dementia Rating Scale, Digit Span, Corsi Block-Tapping Task, Token Test, Rey Auditory Verbal Learning Test (RAVLT), Frontal Assessment Battery, and Tower of London test, all validated for Brazilian Portuguese and adapted for educational level.

Clinical and anthropometric data, including hypertension, dyslipidemia, diabetes mellitus (DM), and smoking status (≥100 cigarettes lifetime), were collected from medical records and interviews. Anthropometric measurements (BMI, abdominal, calf, and hip circumferences) and gait speed (4-meter walk test) were standardized. BMI classification followed Lipschitz (1994): underweight (≤22 kg/m^2^), normal weight (22.1–26.9 kg/m^2^), and overweight (≥27 kg/m^2^). Body composition was evaluated by DXA (Hologic Discovery W, software 3.3.0.1; 2011), measuring total fat percentage, android fat percentage, fat mass index (kg/m^2^), and appendicular lean mass (kg/m^2^), according to International Society for Clinical Densitometry guidelines.

The specific thresholds and cut-offs used in assessments are available upon request and were omitted from the manuscript due to space constraints and for clarity.

### Molecular biology and relative leukocyte telomere length analysis

Peripheral blood samples were collected, and DNA extraction was performed using the saline method. Relative leukocyte telomere length (LTL) was measured via quantitative real-time PCR (qPCR), calculating the telomere/single-copy gene (T/S) ratio (Cawthon, 2002). All qPCR analyses were performed in triplicate and repeated twice, with intra- and inter-assay coefficients of variation evaluated to ensure measurement reliability (coefficients of variation <5 and < 7%, respectively). APOE genotype was determined using allele-specific fluorophore-labeled probes (TaqMan assay).

### Statistical methods

Statistical analysis was performed using IBM SPSS Statistics 20.0. Data distribution normality was tested using the Shapiro–Wilk test. Normally distributed variables were analyzed with one-way ANOVA followed by Bonferroni post-hoc correction; non-normal data underwent Kruskal-Wallis and Mann–Whitney U tests with Bonferroni correction. Categorical variables were compared using Pearson’s chi-square or Fisher’s exact test. Pearson’s or Spearman’s correlation analyses evaluated associations between LTL and continuous variables. Multiple linear regression was performed to identify independent predictors of LTL, adjusting for potential confounders identified in univariate analysis (*p* < 0.20). The regression model accounted for assumptions of linearity, normality, independence, homoscedasticity, and multicollinearity. The final model included cognitive group, age, sex, educational level, hypertension, dyslipidemia, BMI, and calf circumference, also considering potential confounding effects. Interaction analyses evaluated potential modifying effects of cognitive status and APOE genotype on LTL association. To minimize type I error, *p*-values were adjusted using Bonferroni corrections for multiple comparisons.

No formal sample size calculation was performed prospectively; instead, all eligible patients referred during the study period were included, yielding a total of 90 participants equally distributed among CU, MCI, and AD groups. A post-hoc power analysis indicated sufficient power (assuming a large effect size, *d* = 0.8) to detect statistically significant differences. To minimize bias, blinding was implemented, and neuropsychological and geriatric evaluators were unaware of the participants’ group assignments. Standardized protocols were rigorously followed to reduce measurement and classification biases.

The primary outcome was to assess the relationship between cognitive impairment, body composition, and cardiovascular risk factors, including diabetes mellitus, hypertension, dyslipidemia, and smoking status, as well as genetic factors (APOE polymorphisms) and LTL among the three cognitive groups. Potential confounders such as age, sex, educational level, and comorbid conditions were recorded and compared between groups.

## Results

### Enrollment, demographics, and baseline clinical characteristics

**Figure 1:**
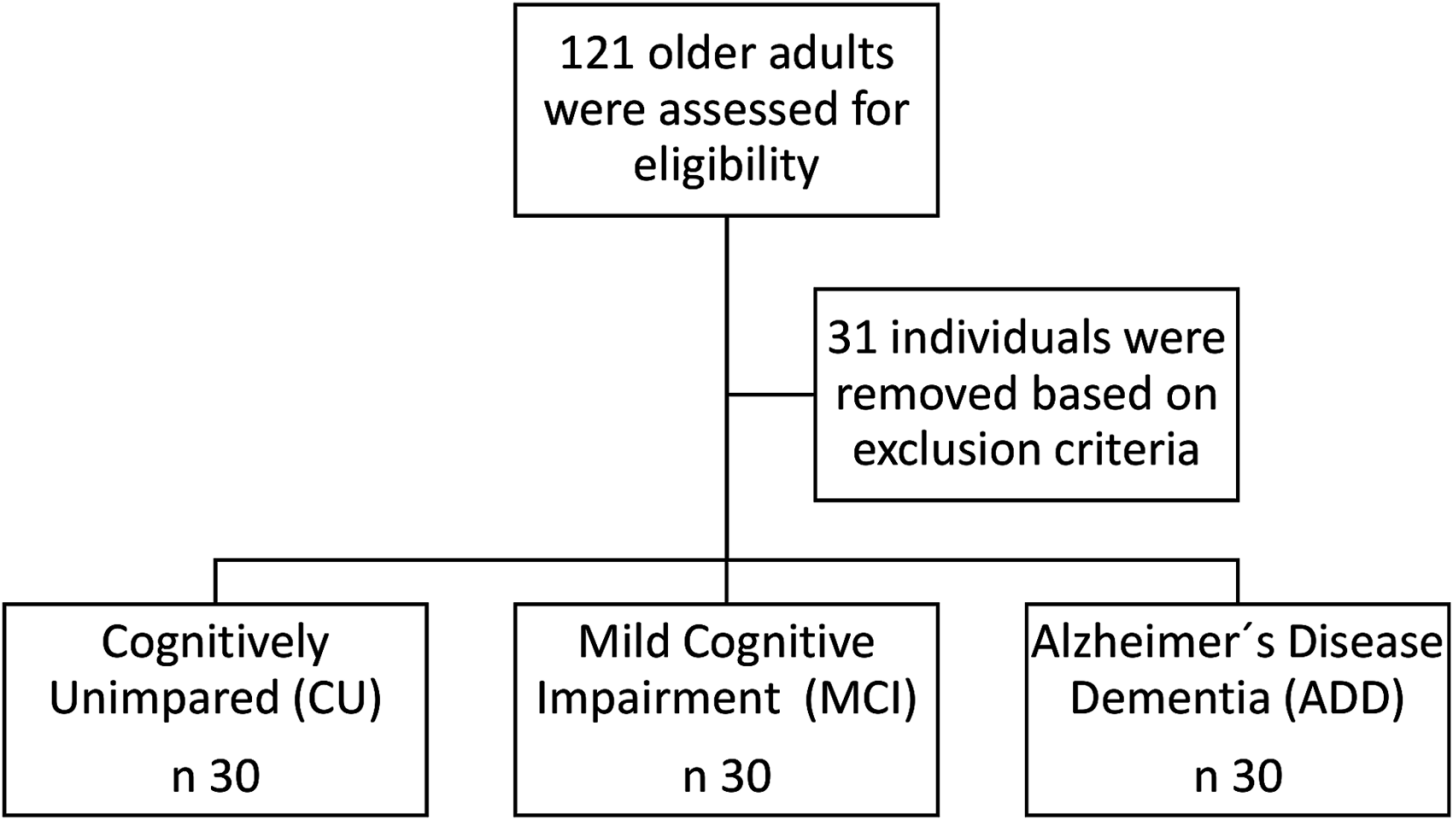
Flowchart illustrating participant recruitment and selection process. Out of 121 older adults initially assessed for eligibility, 31 were excluded based on pre-specified exclusion criteria, resulting in a final sample of 90 participants equally allocated to cognitively unimpaired (CU), mild cognitive impairment (MCI), and Alzheimer’s disease (AD) groups.

**Figure 2:**
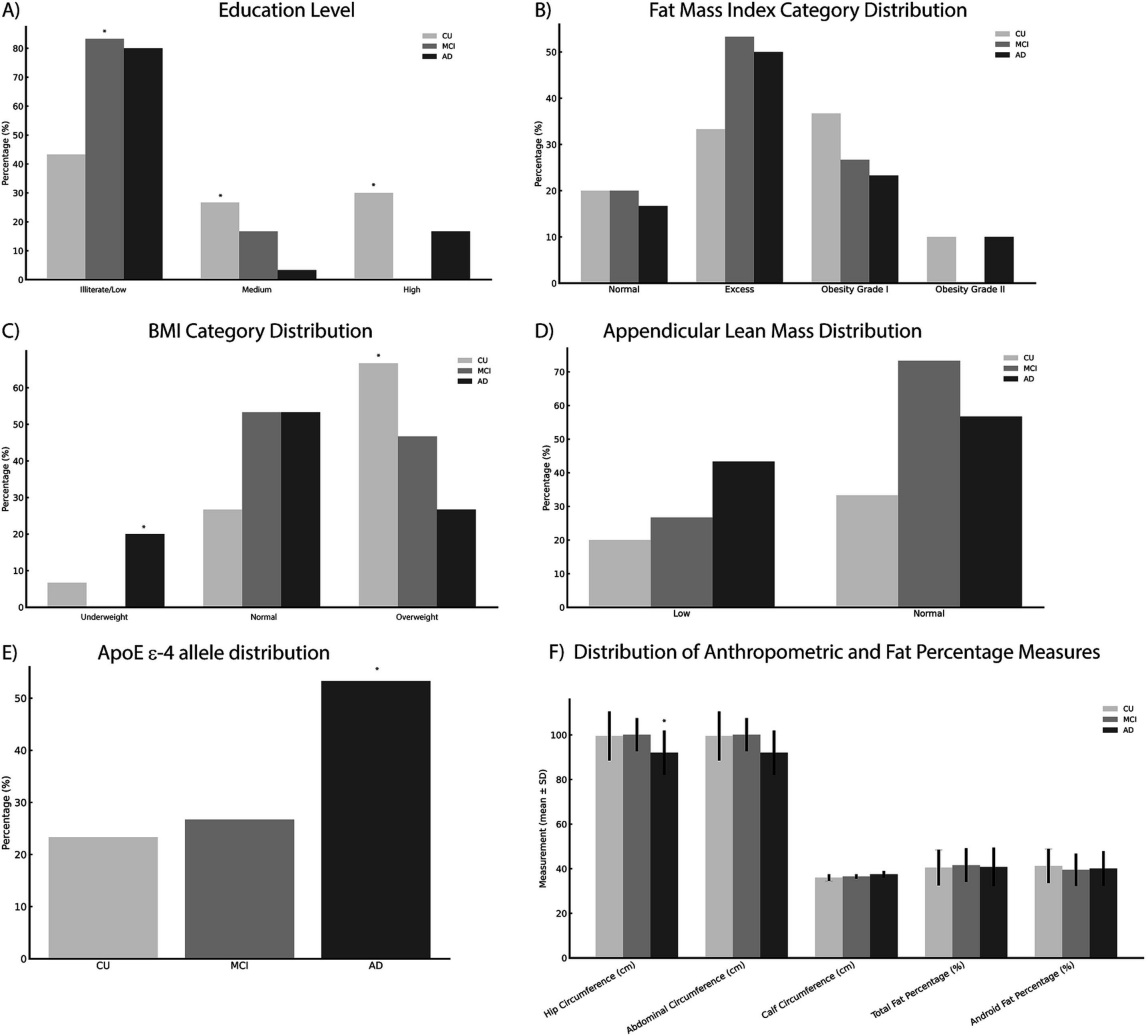
Baseline demographic, clinical, anthropometric, and genetic characteristics summarized across cognitive groups. Data include **(A)** education level, **(B)** fat mass index categories, **(C)** body mass index (BMI), **(D)** appendicular lean mass, **(E)** APOE ε4 allele prevalence, and **(F)** anthropometric measurements (hip, calf, and abdominal circumferences, and fat percentages). Statistically significant differences (*p < 0.05) between groups were observed for age, educational attainment, APOE ε4 allele prevalence, BMI categories, and hip circumference. Statistical comparisons were performed using the Chi-square test, Fisher’s exact test, and the Kruskal–Wallis or One-way ANOVA tests as appropriate. Categorical variables are expressed as frequencies (percentages) and continuous variables as mean ± standard deviation (SD). BMI, body mass index; SD, standard deviation.

During the study period, 121 older adults were initially assessed for eligibility. After excluding 31 individuals based on pre-specified exclusion criteria, the final sample consisted of 90 participants, equally distributed into cognitively unimpaired (CU), mild cognitive impairment (MCI), and Alzheimer’s disease (AD) groups (Figure 1). Most participants were female (68.9%), with a median age of 77.5 years and a mean educational level of 4.56 years. The AD group had significantly older participants compared to CU and MCI groups (*p* = 0.024). Educational attainment was significantly lower in the MCI and AD groups compared to the CU group (*p* = 0.017). No significant gender differences were found across groups. The prevalence of type 2 diabetes mellitus and hypertension was higher in the MCI group, although these differences were not statistically significant among groups. Cardiovascular risk stratification, performed using standardized criteria (Framingham Risk Score categories: low, intermediate, high, very high), did not differ significantly among groups; however, data for cardiovascular risk stratification were incomplete for five participants. The prevalence of the APOE ε4 allele was significantly higher in the AD group compared to the CU and MCI groups (*p* = 0.008) (Table 1 and Figure 2).

**Table 1:** Demographic, clinical, and anthropometric characteristics of study participants stratified by cognitive status: cognitively unimpaired (CU), mild cognitive impairment (MCI), and Alzheimer’s disease (AD).

Variable	Total (*n* = 90)	CU (*n* = 30)	MCI (*n* = 30)	ADD (*n* = 30)	*p*-value
Sex, Female, n (%)	62 (68.9%)	22 (73.3%)	21 (70.0%)	19 (63.3%)	0.696^2^
Age, years, Median (IQR)	77.5 (73.0–81.0)	74.0 (71.0–78.2)	78.6 (73.0–81.2)	79.5 (75.0–83.5)	**0.006** ^**3**^
Smoking Status, n (%)	23 (25.6%)	5 (16.7%)	11 (36.7%)	7 (23.3%)	0.195^1^
Diabetes Mellitus, n (%)	26 (28.9%)	9 (30.0%)	10 (33.3%)	7 (23.3%)	0.077^1^
Hypertension, n (%)	64 (71.1%)	20 (66.7%)	26 (86.7%)	18 (60.0%)	0.060^1^
Dyslipidemia, n (%)	41 (45.6%)	19 (63.3%)	13 (43.3%)	9 (30.0%)	**0.033** ^**1**^

Data are presented as median and interquartile range (IQR) for continuous variables or frequencies (%) for categorical variables. Statistical significance was determined using Chi-square test (^1^), Fisher’s exact test (^2^), and Kruskal–Wallis test (^3^), with significance set at *p* < 0.05. BMI, Body Mass Index. Bold values indicate statistically significant differences between groups (*p* < 0.05).

### Telomere length and cognitive impairment

**Figure 3:**
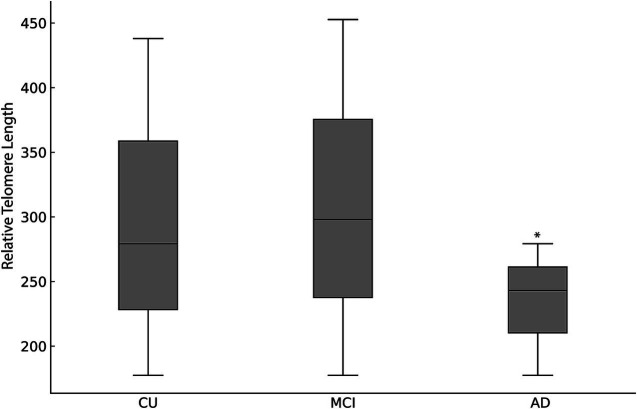
Relative leukocyte telomere length expressed as median (interquartile range [IQR]), across cognitive status groups: CU, MCI and AD. Significant telomere shortening was observed in the AD group compared to CU and MCI groups (*p* < 0.001, Kruskal–Wallis test). Differences between CU and MCI groups were not statistically significant.

Relative leukocyte telomere length differed significantly among cognitive groups. Participants in the AD group demonstrated significantly shorter telomeres compared to the CU group (*p* = 0.006) and the MCI group (*p* = 0.032), while no significant difference was found between the CU and MCI groups (Figure 3).

### Body composition and telomere length

BMI categories significantly varied across groups, with a higher frequency of overweight individuals in the CU group compared to AD and MCI groups (*p* = 0.043). No significant differences were observed regarding fat mass index, hip circumference, or calf circumference across cognitive groups (Figure 2).

Correlation analyses between telomere length and anthropometric or body composition variables revealed no significant associations within or across cognitive groups, except for a positive correlation between hypertension and telomere length specifically within the MCI group (*p* = 0.035). No significant associations were observed between telomere length and BMI categories, fat mass index, or other cardiovascular risk factors (all *p* > 0.05) (Supplementary Tables 1, 2).

### Multivariate regression analysis

**Figure 4:**
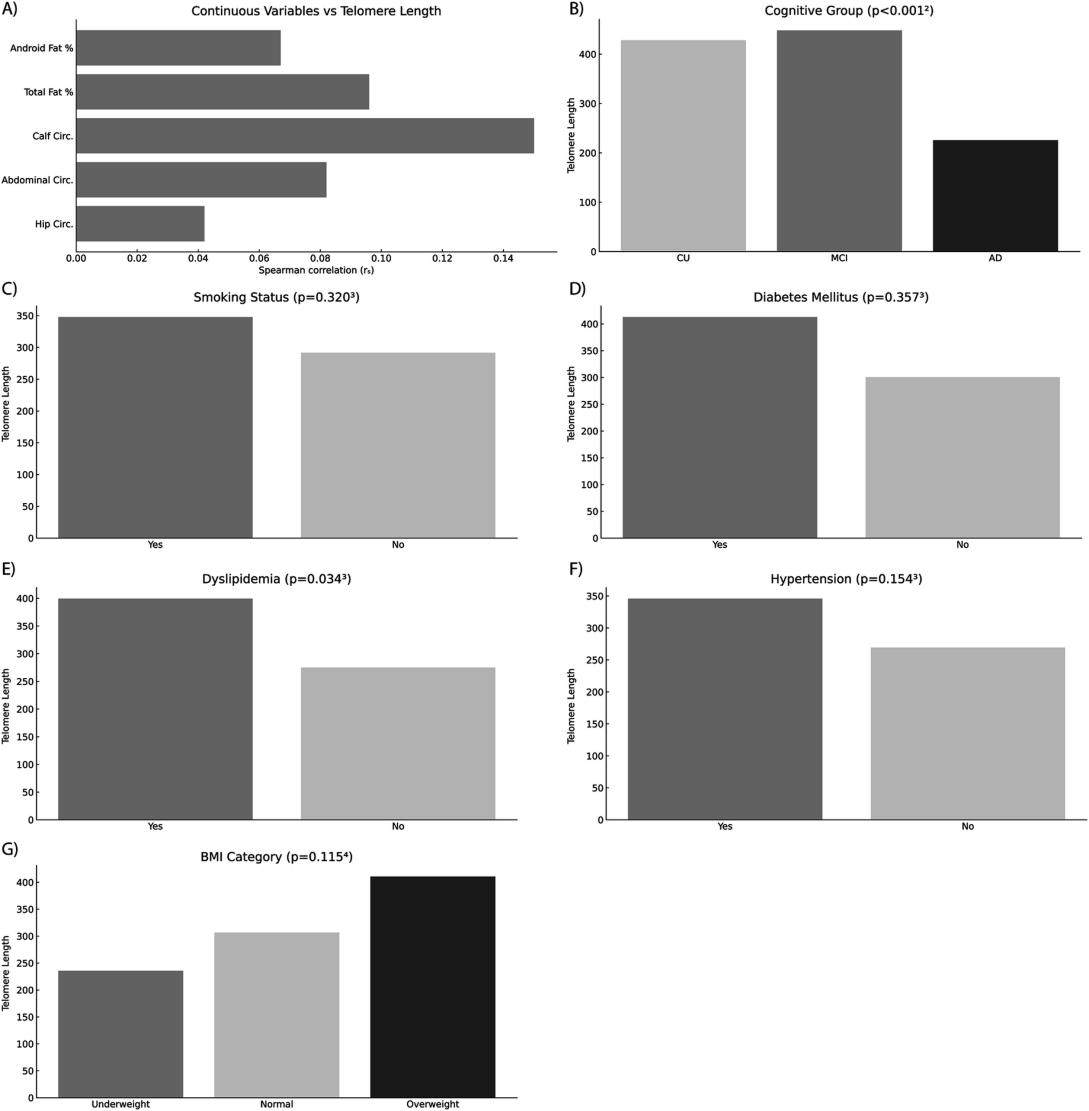
Multivariate regression analysis assessing predictors of relative leukocyte telomere length. The figure displays the relationships between telomere length and various clinical, anthropometric, and demographic variables evaluated in the regression model, divided into distinct categories: **(A)** Anthropometric and body composition parameters; **(B)** Cognitive status group (CU, cognitively unimpaired; MCI, mild cognitive impairment; AD, Alzheimer’s disease); **(C)** Smoking status; **(D)** Diabetes mellitus; **(E)** Dyslipidemia; **(F)** Hypertension; and **(G)** BMI category. Alzheimer’s disease (AD) was identified as a significant independent predictor of shorter telomeres compared to CU and MCI groups (*p* < 0.05). Other variables tested, including anthropometric and body composition parameters, smoking status, diabetes mellitus, dyslipidemia, hypertension, and BMI categories, did not show statistically significant associations with telomere length. Statistical significance was evaluated using Spearman’s rank correlation coefficient (r_s_), Mann–Whitney U test, Kruskal–Wallis test, and Jonckheere–Terpstra test as appropriate. CU, cognitively unimpaired; MCI, mild cognitive impairment; BMI, Body Mass Index; r_s_, Spearman’s rank correlation coefficient.

**Figure 5:**
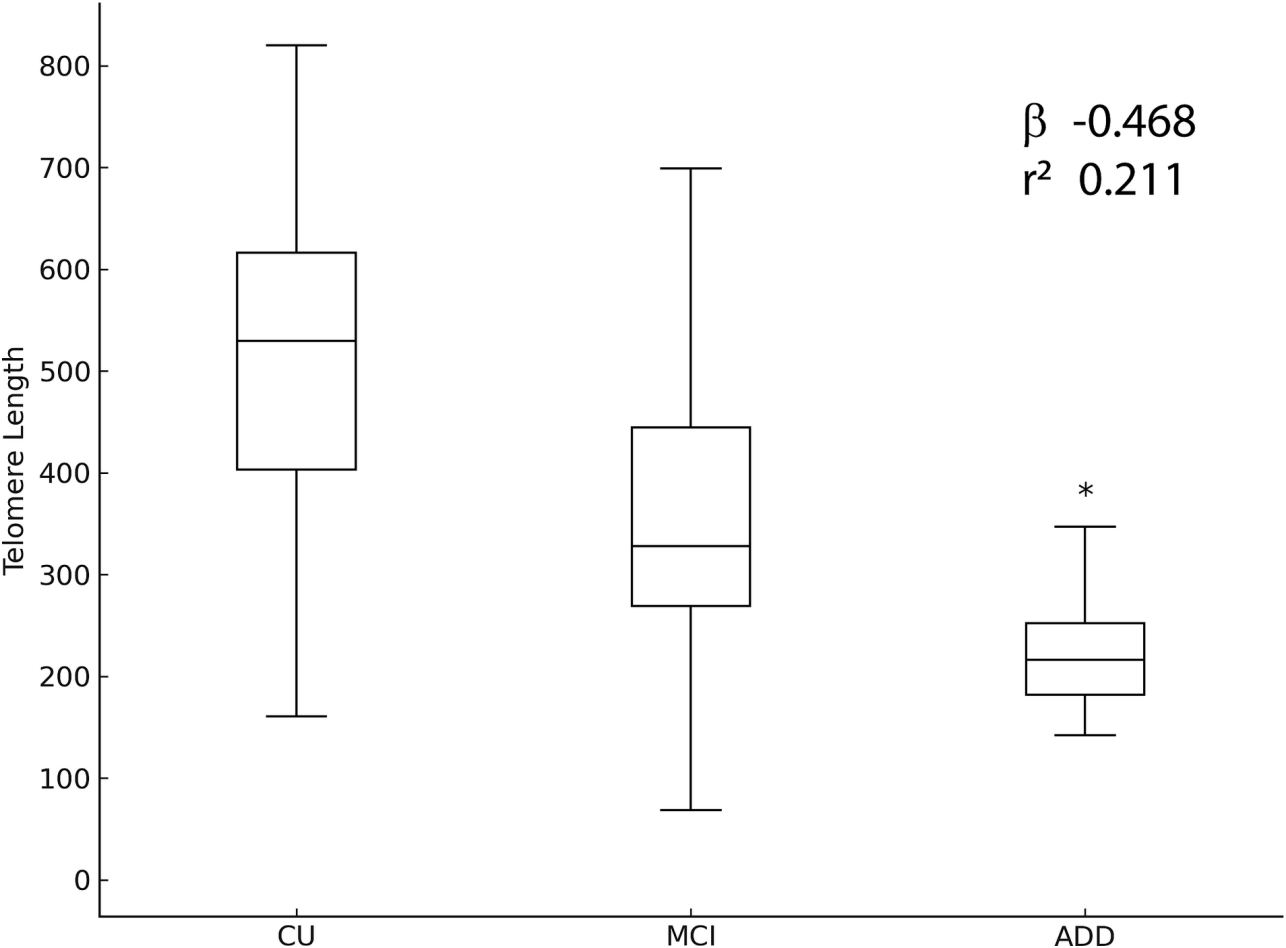
Multivariate regression analysis illustrating the association between cognitive status and relative leukocyte telomere length. Adjusted *β*-coefficients and corresponding 95% confidence intervals (CI) indicate significantly shorter telomere lengths in the Alzheimer’s disease group compared to other groups. The regression model was adjusted for age, sex, educational level, BMI, hypertension, dyslipidemia, calf circumference, and body composition. Statistical significance was set at *p* < 0.05.

Multiple linear regression analysis assessed independent predictors of LTL, incorporating cognitive group, age, sex, educational level, body composition and cardiovascular risk factors as covariates (Figure 4). The regression model explained 25.3% of the variance in LTL (*r*^2^ = 0.211, *β*−0.468, 95% CI [−283,369 to −121,587], *p* < 0.001) (Figure 5). Cognitive group was the only significant predictor, with the AD group presenting significantly shorter telomeres compared to CU and MCI groups (*β* = −0.34, 95% CI [−0.56 to −0.12], *p* = 0.003). None of the cardiovascular risk factors or body composition variables reached statistical significance as independent predictors of LTL.

Interaction analyses between APOE genotype and cognitive status were conducted, revealing no significant interactions influencing telomere length (interaction *p* > 0.05). Additionally, no significant differences in demographic variables, including education and BMI, were identified between APOE ε4 carriers and non-carriers, indicating limited confounding effects by these variables. At this point, it is important to note that although a post-hoc power analysis indicated adequate statistical power (>80%) to detect significant group differences in telomere length, given the observed large effect size (*d* = 0.8), caution is warranted when interpreting negative findings related to cardiovascular risk factors and body composition, due to the limited sample size and potential insufficient power to detect smaller effects. All post-hoc comparisons were adjusted using Bonferroni corrections to minimize the risk of Type I errors due to multiple testing.

## Discussion

In this study, we identified significant associations between relative leukocyte telomere length and cognitive impairment among older adults, demonstrating shorter telomeres in individuals with Alzheimer’s disease compared to those cognitively unimpaired and those with mild cognitive impairment. These findings reinforce the growing body of evidence linking telomere attrition to advanced cognitive impairment and the neuropathological mechanisms underlying AD. Shortened telomeres are known to activate cellular senescence pathways and inflammatory cascades, potentially contributing to the pathological hallmarks of AD, such as amyloid-beta plaques, tau aggregation, and neurodegeneration (Guo et al., 2022; Müezzinler et al., 2013; Yu and Koh, 2022; López-Otín et al., 2023; Blackburn et al., 2015; Rossiello et al., 2022; Fani et al., 2019; Vaiserman and Krasnienkov, 2021; Yu et al., 2021; Rodríguez-Fernández et al., 2022; Crocco et al., 2023; Wang et al., 2018; Zekry et al., 2010). Contrary to initial expectations, cardiovascular risk factors, including diabetes mellitus, hypertension, dyslipidemia, and anthropometric indicators of cardiovascular risk, were not independently associated with telomere length across cognitive groups. These findings differ from previous literature demonstrating associations between cardiovascular risk factors and shorter telomeres (Demissie et al., 2006; Samani et al., 2001; Epel et al., 2004; Schneider et al., 2022; Cheng et al., 2020; Yin and Pickering, 2023; Fragkiadaki et al., 2025). Possible explanations for our findings include modest sample size, insufficient statistical power to detect subtle associations, and confounding effects from medication use, lifestyle behaviors, and genetic variability in the study population. Our results thus highlight the complexity and heterogeneity of mechanisms involved in cellular aging and cognitive decline (López-Otín et al., 2023; Schneider et al., 2022; Sahin et al., 2010).

Additionally, we observed a higher prevalence of the APOE ε4 allele in participants with AD, consistent with its well-established role as a genetic risk factor for AD (Corder et al., 1993; Farrer et al., 1997). However, interaction analyses revealed no significant modifying effect of APOE genotype on telomere length associations, suggesting that telomere attrition in AD may occur independently of APOE ε4-related mechanisms.

A key strength of our study includes the rigorous characterization of cognitive status through comprehensive neuropsychological evaluations alongside detailed assessments of anthropometric, cardiovascular, and genetic parameters uniquely validated within an elderly Brazilian population. This demographic is typically underrepresented in aging research, contributing novel insights into regional variability in telomere dynamics and cognitive impairment relationships.

Several limitations warrant consideration. The cross-sectional design restricts our capacity to infer causality or temporal relationships between telomere shortening and cognitive impairment progression. Although resource and logistical constraints influenced our design choice, longitudinal studies remain necessary to elucidate these associations’ dynamic nature. Moreover, although our sample size provided adequate statistical power for detecting large effect sizes in telomere length differences among cognitive groups, it may have been insufficient to detect smaller yet clinically relevant associations with cardiovascular risk factors. Additionally, the qPCR method used to measure telomere length calculates an average telomere/single-copy gene ratio rather than directly evaluating absolute telomere size, including short telomeres, potentially limiting insights into specific pathological mechanisms.

Future research directions should include larger-scale, longitudinal cohort studies employing methods capable of detecting critically short telomeres and exploring mechanistic links between telomere biology, cellular senescence, chronic inflammation, and neurodegenerative processes. Interventional studies targeting lifestyle modifications or pharmacological strategies to preserve telomere integrity may also yield important insights and potential therapeutic avenues.

In conclusion, our study provides evidence of a significant association between leukocyte telomere shortening and Alzheimer’s disease, independent of traditional cardiovascular risk factors and APOE ε4 genotype. These findings underscore the importance of telomere biology in neurodegenerative disorders and highlight the potential role of telomere length as part of a broader biomarker panel for identifying older adults at increased risk of cognitive decline and AD.

## Data Availability

The raw data supporting the conclusions of this article will be made available by the authors, without undue reservation.

## Supplementary material

SUPPLEMENTARY TABLE 1 Associations between relative leukocyte telomere length and cardiovascular risk factors stratified by cognitive status: cognitively unimpaired (CU), mild cognitive impairment (MCI), and Alzheimer's disease (AD). Relative telomere length (expressed as T/S ratio; mean ± standard deviation) comparisons were conducted for cardiovascular variables including type 2 diabetes mellitus, hypertension, dyslipidemia, and cardiovascular risk stratification (low, intermediate, high, very high). Statistical significance was assessed using Spearman’s rank correlation coefficient, with significance indicated by *p*-values < 0.05. Notably, a significant association was observed only between hypertension and telomere length in the MCI group (*p* < 0.001). SD, Standard Deviation; N, number of participants per group.

SUPPLEMENTARY TABLE 2 Correlation analysis of relative leukocyte telomere length with anthropometric and body composition parameters stratified by cognitive status: cognitively unimpaired (CU), mild cognitive impairment (MCI), and Alzheimer’s disease (AD). Continuous variables (calf, abdominal, and hip circumferences; total and android fat percentages) are expressed as Pearson’s correlation coefficients (r) with corresponding *p*-values. Categorical variables, including fat mass index and appendicular lean mass categories, are presented as mean ± standard deviation (SD). Analysis indicates no statistically significant correlation between telomere and the evaluated continuous or categorical body composition variables across cognitive groups. BMI, Body Mass Index; N/A, data not available.
