# Supplementary material for: Leukocyte telomere attrition in cognitive decline: associations with APOE genotype and cardiovascular risk factors
Source: Front Aging Neurosci. 2025 Apr 15;17:1557016. doi: 10.3389/fnagi.2025.1557016 (PMC12037525; doi:10.3389/fnagi.2025.1557016)
Supplement: SUPPLEMENTARY TABLE 2 — Correlation analysis of relative leukocyte telomere length with anthropometric and body composition parameters stratified by cognitive status: cognitively unimpaired (CU), mild cognitive impairment (MCI), and Alzheimer’s disease (AD). Continuous variables (calf, abdominal, and hip circumferences; total and android fat percentages) are expressed as Pearson’s correlation coefficients (r) with corresponding p-values. Categorical variables, including fat mass index and appendicular lean mass categories, are presented as mean ± standard deviation (SD). Analysis indicates no statistically significant correlation between telomere and the evaluated continuous or categorical body composition variables across cognitive groups. BMI, Body Mass Index; N/A, data not available. [file Data_Sheet_2.PDF]

Supplementary Table 1

Variable	Control (N = 30)	MCI (N = 30)	AD (N = 30)	p-value (Control)	p-value (MCI)	p-value (AD)
Smoking	346.26 ± 189.54	505.62 ± 164.32	275.40 ± 86.99	0.368[1]	0.209[1]	0.332[1]
Diabetes Type 2	424.64 ± 268.53	467.69 ± 196.70	263.59 ± 98.74	0.845[1]	0.644[1]	0.541[1]
Dyslipidemia	438.21 ± 245.55	516.33 ± 183.03	278.39 ± 107.28	0.995[1]	0.780[1]	0.206[1]
Hypertension	415.50 ± 238.49	477.73 ± 172.30	246.51 ± 107.71	0.420[1]	**<0.001[1]**	0.821[1]
**Cardiovascular Risk**						
Low	N/A	N/A	N/A			
Intermediate	460.03 ± 328.35	N/A	N/A			
High	468.07 ± 260.72	457.11 ± 173.53	238.20 ± 94.39	0.552[2]	0.596[1]	0.543[1]
Very High	347.58 ± 118.72	418.06 ± 179.40	203.96 ± 7.51			

Supplementary Table 2

Variable	Control (N = 30)	MCI (N = 30)	AD (N = 30)
**Continuous Variables**			
**Calf Circumference**	r = 0.160 (p = 0.393)	r = 0.023 (p = 0.904)	r = 0.081 (p = 0.672)
**Abdominal Circumference**	r = 0.012 (p = 0.951)	r = –0.318 (p = 0.086)	r = 0.246 (p = 0.191)
**Hip Circumference**	r = 0.005 (p = 0.979)	r = –0.087 (p = 0.649)	r = 0.239 (p = 0.204)
**Total Fat Percentage**	r = 0.130 (p = 0.494)	r = 0.015 (p = 0.939)	r = 0.017 (p = 0.928)
**Android Fat Percentage**	r = 0.086 (p = 0.650)	r = –0.007 (p = 0.969)	r = 0.040 (p = 0.836)
**Categorical Variables**			
**Fat Mass Index**			
Normal Fat Mass	451.27 ± 245.26	318.38 ± 210.51	262.10 ± 122.02
Excess Fat Mass	391.31 ± 254.28	480.99 ± 186.21	220.54 ± 57.06
Obesity Grade I	454.44 ± 268.57	446.75 ± 137.93	258.36 ± 127.58
Obesity Grade II	539.52 ± 185.75	N/A	N/A
**Appendicular Lean Mass**			

Low	434.11 ± 216.99	467.64 ± 160.12	210.48 ± 71.68
Normal	439.75 ± 258.90	444.06 ± 184.30	268.03 ± 137.85
**Body Mass Index**			
Underweight	509.96 ± 439.53	N/A	201.47 ± 89.97
Normal Weight	385.60 ± 155.65	466.98 ± 187.97	248.81 ± 92.96
Overweight	452.42 ± 267.35	436.40 ± 160.46	262.89 ± 119.34
